# Supplementary figures and images for: Cancer Type Classification in Liquid Biopsies Based on Sparse Mutational Profiles Enabled through Data Augmentation and Integration
Source: Life (Basel). 2021 Dec 21;12(1):1. doi: 10.3390/life12010001 (PMC8780455; doi:10.3390/life12010001)

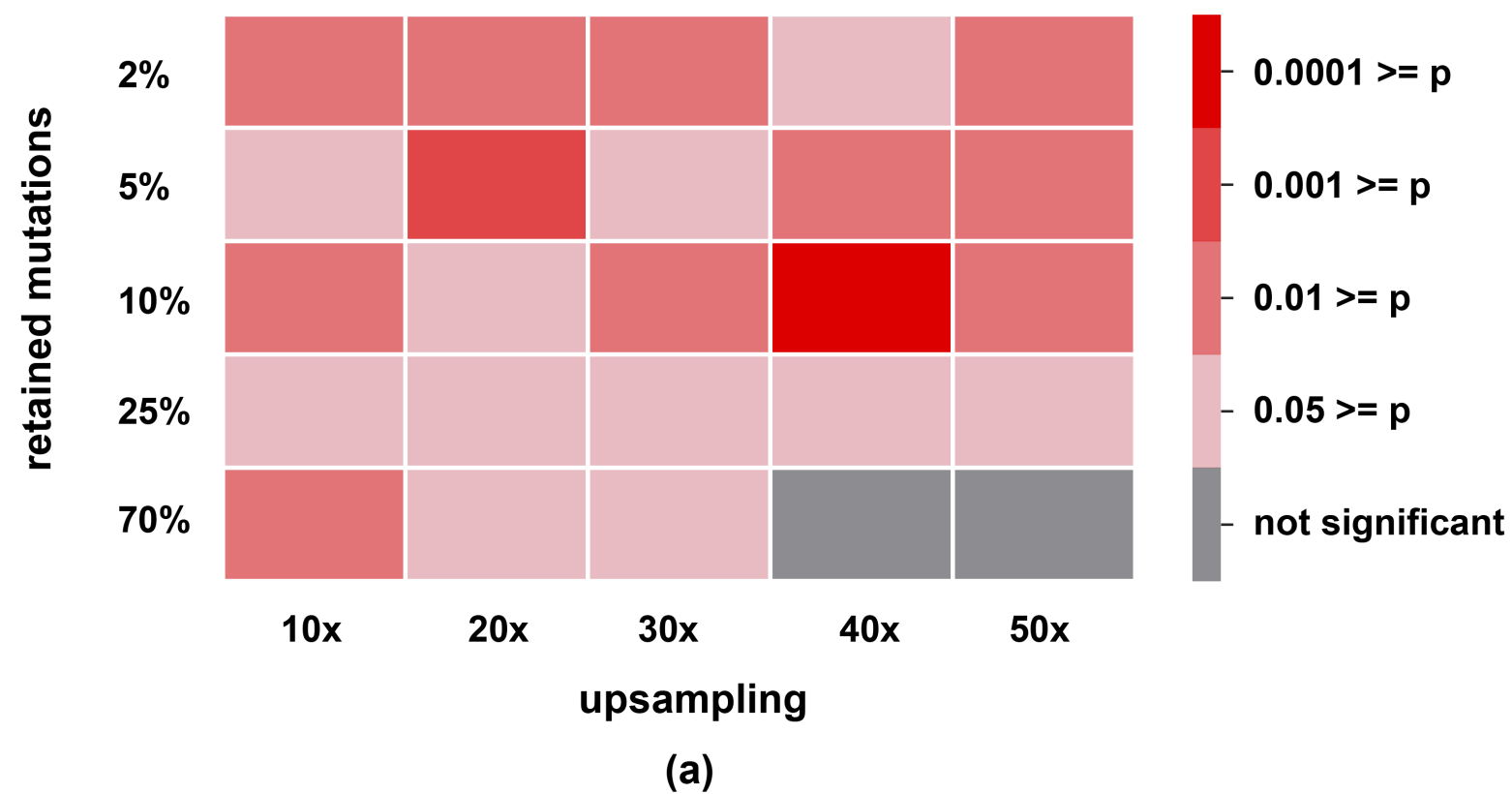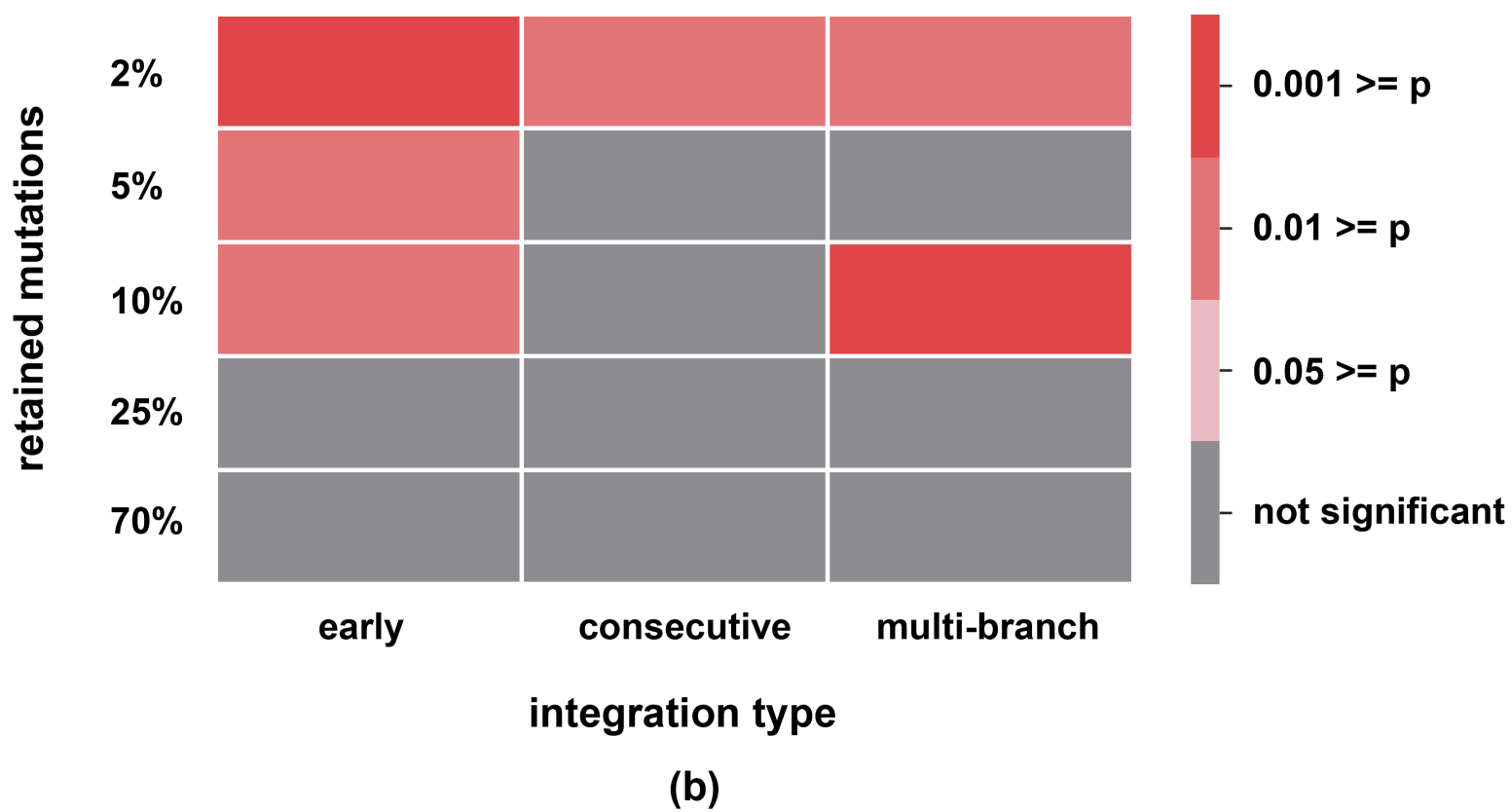

Supplement: Supplementary file 1 [file life-12-00001-s001.zip › Supplementary_material/S5_Fig.pdf]

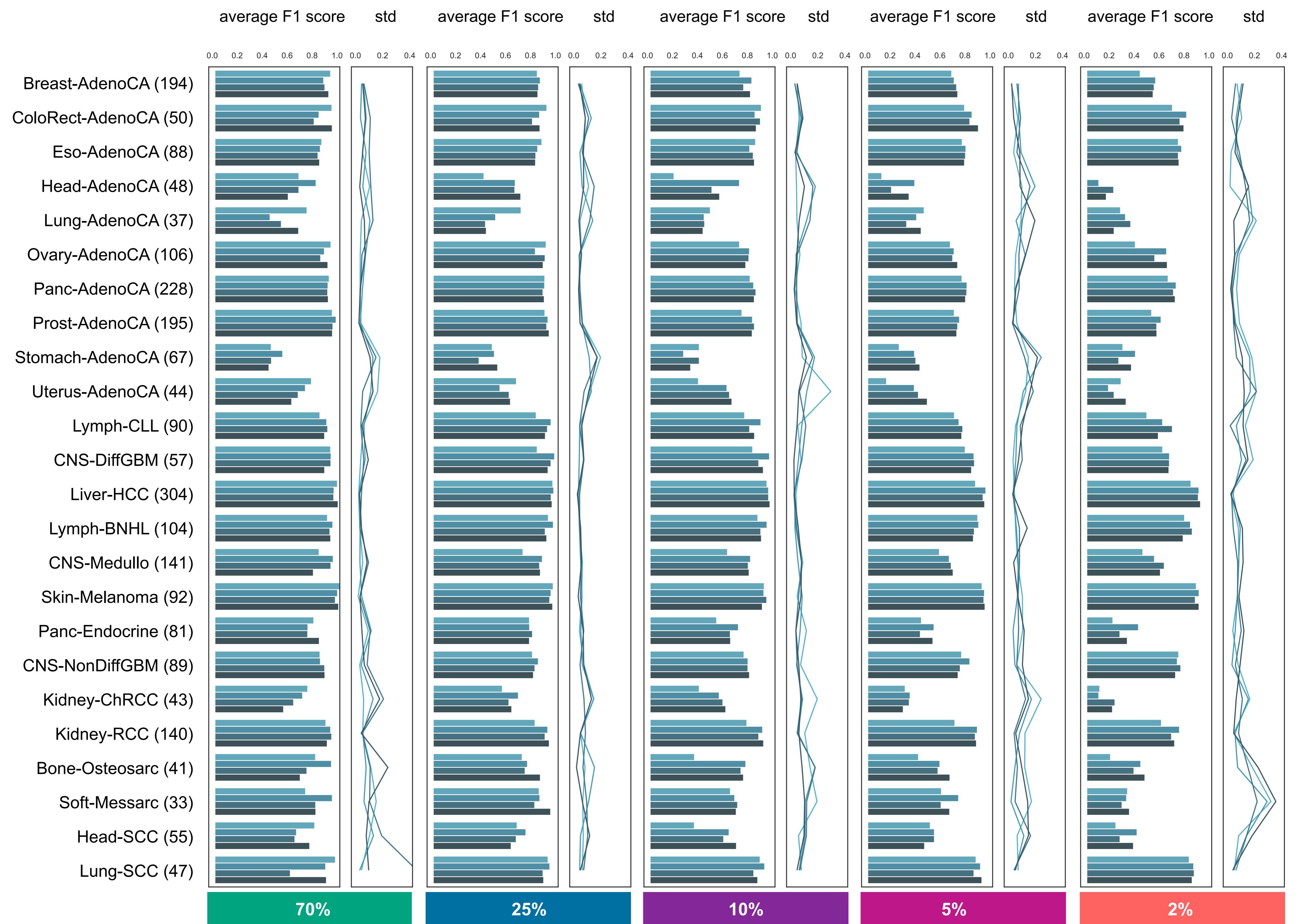

■ no data integration  
■ early integration  
■ consecutive integration  
■ multi-branch integration

Supplement: Supplementary file 1 [file life-12-00001-s001.zip › Supplementary_material/S4_Fig.pdf]

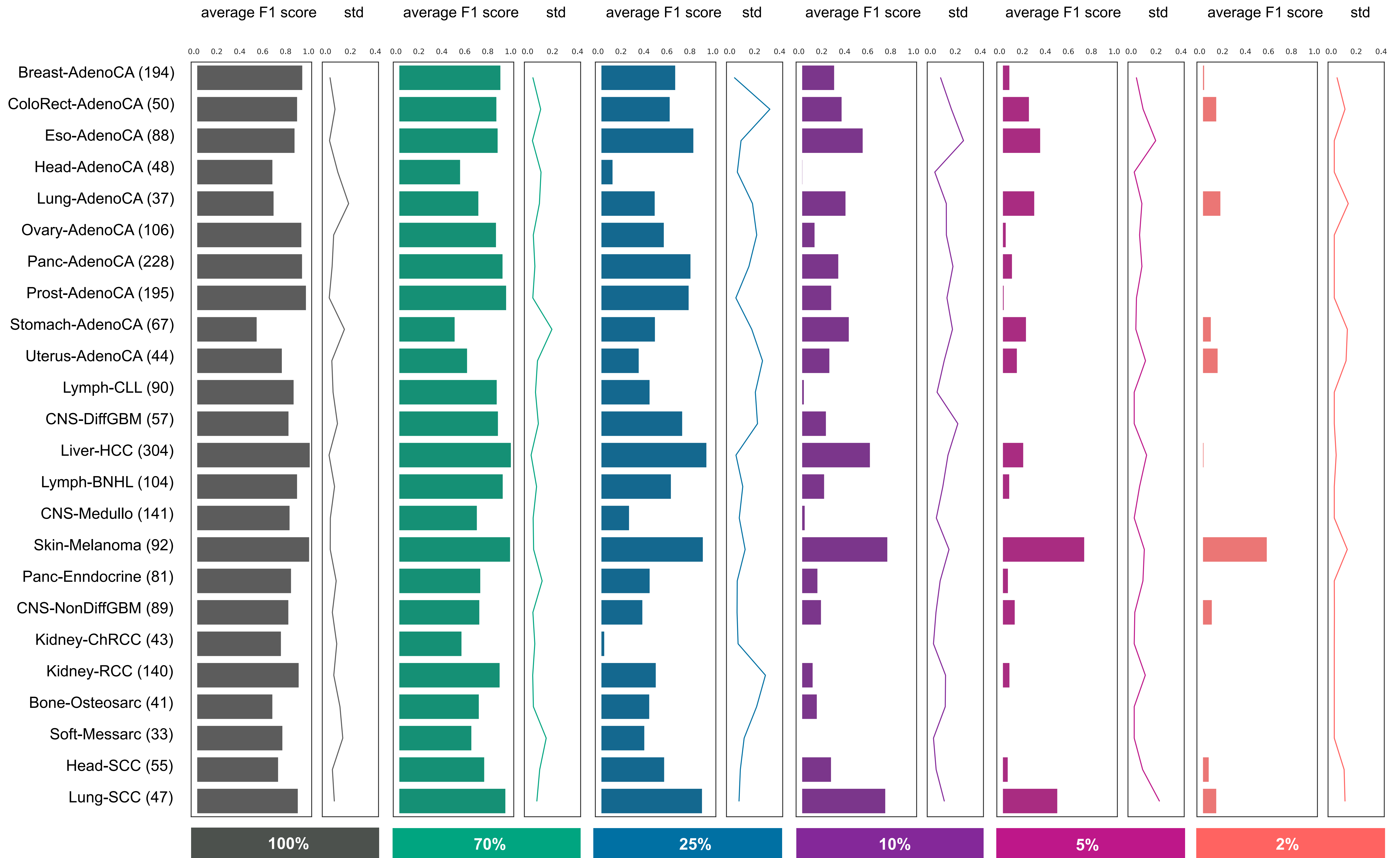

Supplement: Supplementary file 1 [file life-12-00001-s001.zip › Supplementary_material/S1_Fig.pdf]

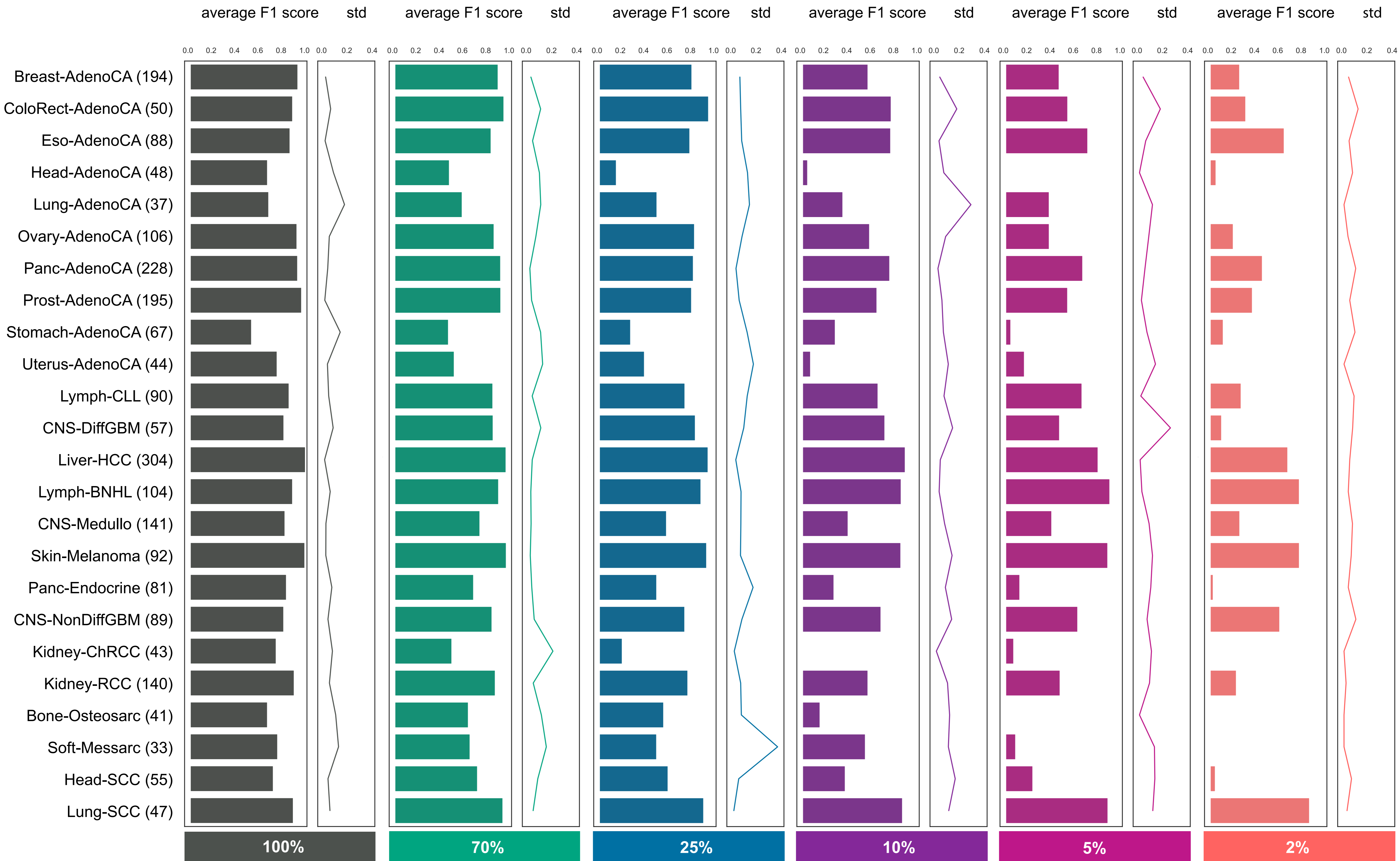

Supplement: Supplementary file 1 [file life-12-00001-s001.zip › Supplementary_material/S2_Fig.pdf]

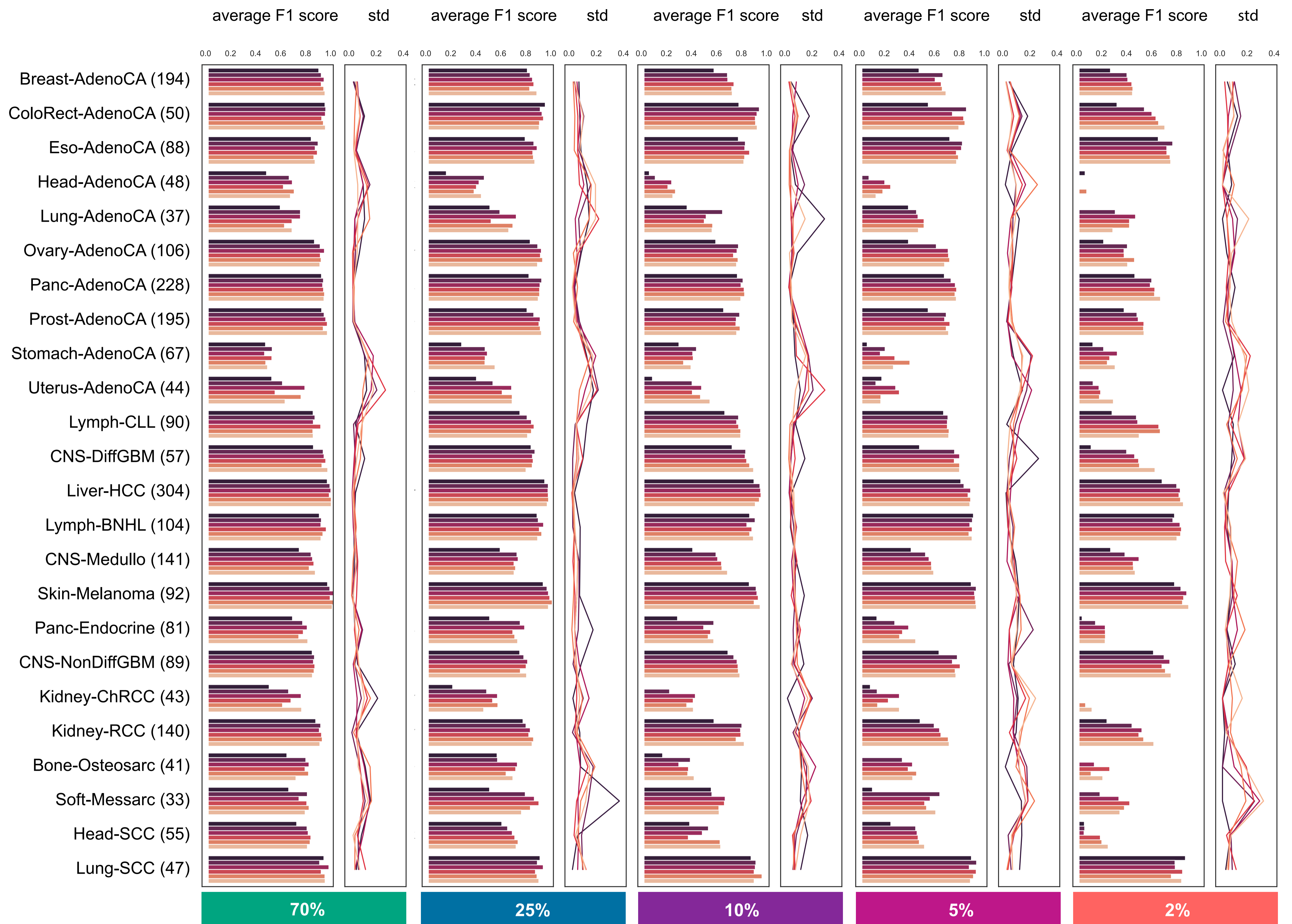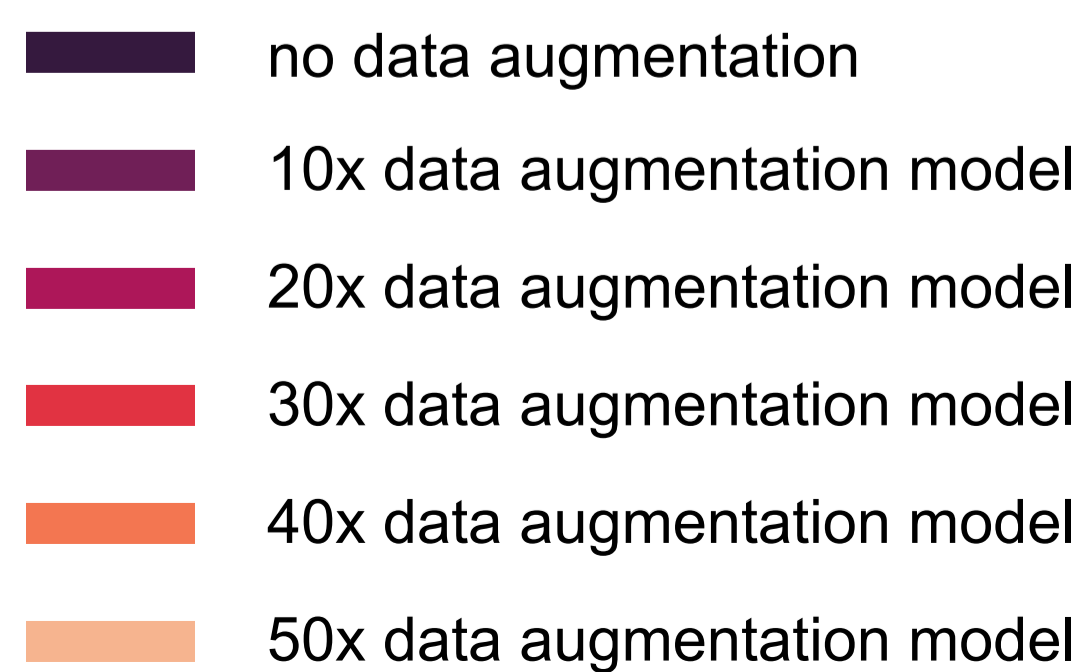

Supplement: Supplementary file 1 [file life-12-00001-s001.zip › Supplementary_material/S3_Fig.pdf]
